# Oxidative Stress in Neurodegenerative Diseases: Mechanisms and Therapeutic Perspectives

**DOI:** 10.1155/2011/467180

**Published:** 2011-11-24

**Authors:** Ailton Melo, Larissa Monteiro, Rute M. F. Lima, Diêgo M. de Oliveira, Martins D. de Cerqueira, Ramon S. El-Bachá

**Affiliations:** ^1^National Council for Scientific and Technological Development (CNPq), Faculty of Medicine, Federal University of Bahia, 40820-020 Salvador, BA, Brazil; ^2^CAPES, Faculty of Medicine, Federal University of Bahia, Campus of Canela, 40820-020 Salvador, BA, Brazil; ^3^CNPq, Institute of Health Sciences, Federal University of Bahia, Campus of Canela, 40110-902 Salvador, BA, Brazil; ^4^Institute of Chemistry, Federal University of Bahia, Campus of Ondina, 40.170-115 Salvador, BA, Brazil

## Abstract

The incidence and prevalence of neurodegenerative diseases (ND) increase with life expectancy. This paper reviews the role of oxidative stress (OS) in ND and pharmacological attempts to fight against reactive oxygen species (ROS)-induced neurodegeneration. Several mechanisms involved in ROS generation in neurodegeneration have been proposed. Recent articles about molecular pathways involved in ROS generation were reviewed. The progress in the development of neuroprotective therapies has been hampered because it is difficult to define targets for treatment and determine what should be considered as neuroprotective. Therefore, the attention was focused on researches about pharmacological targets that could protect neurons against OS. Since it is necessary to look for genes as the ultimate controllers of all biological processes, this paper also tried to identify gerontogenes involved in OS and neurodegeneration. Since neurons depend on glial cells to survive, recent articles about the functioning of these cells in aging and ND were also reviewed. Finally, clinical trials testing potential neuroprotective agents were critically reviewed. Although several potential drugs have been screened in *in vitro* and *in vivo* models of ND, these results were not translated in benefit of patients, and disappointing results were obtained in the majority of clinical trials.

## 1. Introduction

The incidence of neurodegenerative diseases (NDs) increases with extended life expectancy. Age-related degenerative diseases are increasing to epidemic proportions in all industrialized countries. The rise in elderly populations is resulting in an increased incidence of mild cognitive impairment, which is characterized by a less severe (but abnormal) level of cognitive impairment and a high risk for developing dementia. The incidence and prevalence of severe cognitive impairment is also rising. Alzheimer's disease (AD) affects approximately 4.5 million Americans, projected to increase to 11 and to 16 million by 2050; Parkinson's disease (PD) afflicts approximately one million persons in the United States, 60,000 new cases are diagnosed every year, and its incidence is projected to quadruple by 2040 [1]. PD represents a growing socioeconomic burden on societies. In Spain, NDs were included in the Strategic Action on Health, and the Network Centres of Biomedical Research assigned *€* 6862 million to integrate 64 groups and 493 researchers working in this field [2]. 

The neuronal cell oxidative stress (OS) response has an intense interest for neuroscientists because it is a hotspot on neurodegeneration. The generation of reactive oxygen species (ROS) and oxidative damage is believed to be involved in the pathogenesis of neurodegenerative disorders. Biological systems that require oxygen for life are always under various levels of OS. The main ROS involved in neurodegeneration are superoxide anion (O_2_^−^), hydrogen peroxide (H_2_O_2_), and the highly reactive hydroxyl radical (HO^∙^). Furthermore, reactive nitrogen species (RNS) such as nitric oxide (NO) can also damage neurons. The free radical NO can react with O_2_^−^ to produce peroxynitrite (ONOO^−^), a powerful oxidant, which may decompose to form HO^∙^. Oxidative stress results from a misbalance between ROS generation and antioxidant defenses. The widely accepted OS theory of aging postulates that it results from accumulation of oxidative damages. There is evidence that malnutrition, OS, and homocysteine-related vitamins play a role in the pathogenesis of AD [3]. Dietary antioxidant deficiency facilitated the induction of spreading depression in rat brains by the photoactivation of riboflavin, which generates ROS [4]. Oxidative damage has also been associated with pathological neuronal loss in PD [5] and Huntington's disease (HD). Beyond AD, PD, and HD, there are evidences pointing out the important role of OS in other neurodegenerative disorders such as Machado-Joseph disease (MJD) [6]. Therefore, OS provides a crucial link between pathological pathways that occur independently in distinct ND.

The progress in the development of neuroprotective therapies has been hampered by several factors. Without knowing the precise underlying mechanisms of cell death in neurodegenerative disorders, such as PD and AD, it is difficult to define targets for treatment and determine what should be considered as neuroprotective. The development of drugs that modify the progression of ND remains the most important goal in the treatment of these disorders. Drug screening *in vitro* is the first step in drug discovery and development. Once a potential candidate is found, it is tested *in vivo*, in animal models, prior to be submitted to clinical trials. Several drugs have attracted attention as potential neuroprotective agents in the laboratory, but clinical efficacy for neuroprotection remains unproven. This paper intends to review advances made in the study about the role of OS in ND and pharmacological attempts to fight against ROS-induced neurodegeneration.

## 2. Molecular Mechanisms

Although not yet definitive, several possible mechanisms of neurodegeneration have been proposed in the scientific literature. The mitochondrion has been identified as a major source of ROS, and its dysfunction with time appears to contribute to neural decay and ageing [7]. An important feature of this theory is that it creates a possibility for intervention to modify the rate and the conditions of ageing [8]. The mitochondrion is a major site of cellular O_2_^−^ production, mainly derived from Complex I due to partial reduction of NADH-dehydrogenase bound FMN; and from Complex III also due to partial reduction of ubiquinone/ubisemiquinone/ubiquinol, by transfer of one electron to O_2_ [9]. Based in this theory, inhibitors of mitochondrial complexes are currently used to induce OS creating *in vitro* and *in vivo* models of ND, which are useful in drug screening.

Another mitochondrial source of ROS is the enzyme family of monoamine oxidase (MAO; EC 1.4.3.4). These enzymes are bound to the outer mitochondrial membrane and catalyze the oxidation of biogenic amine neurotransmitters such as norepinephrine, dopamine, and 5-hydroxytryptamine (serotonin), but they generate free radicals during their activity. MAO-B catalyzes the oxidation of 1-methyl-4-phenyl-1,2,3,6-tetrahydropyridine (MPTP) to MPP^+^, which inhibits mitochondrial complex I and initiates biochemical, clinical, and neuropathological changes analogous to those observed in idiopathic PD. 2-Amino-6-trifluoromethoxybenzothiazole (riluzole), an Na^+^ channel blocker with antiglutamatergic activity, significantly prevented MPTP-induced reduction in striatal dopamine and 3,4-dihydroxyphenylacetic acid levels in mice [10]. 3-Nitropropionic acid, which blocks succinic dehydrogenase (EC 1.3.99.1) in mitochondrial Complex II, produces pathological changes and cell damages and induces apoptosis in mouse striatal tissue [11]. Therefore, this compound has been used as pharmacological tool in animal models to address some of the metabolic modifications that might underlie central neurodegeneration. The treatment of neuroblastoma cells (SK-N-MC) with low doses of antimycin A1, which is a mitochondrial complex III inhibitor, and 2-deoxy-D-glucose moderately impaired cellular functions by a longer lasting modest OS, reduction of mitochondrial function and reduced intracellular energy levels, simulating a slow chronic effect [12]. In this model, these same authors showed that the *Ginkgo biloba* extract EGb 761 significantly protected cells against reduced glutathione (GSH) depletion. There is a summing knowledge in scientific literature showing that the neurodegeneration in PD is associated with a cascade of events that includes OS, mitochondrial abnormalities, excitotoxity, protein accumulation, inflammation, and ultimately apoptosis [13]. Therefore, preclinical strategies targeted in mitochondrial ROS generation are useful to identify potential drug candidates.

**Figure 1:**
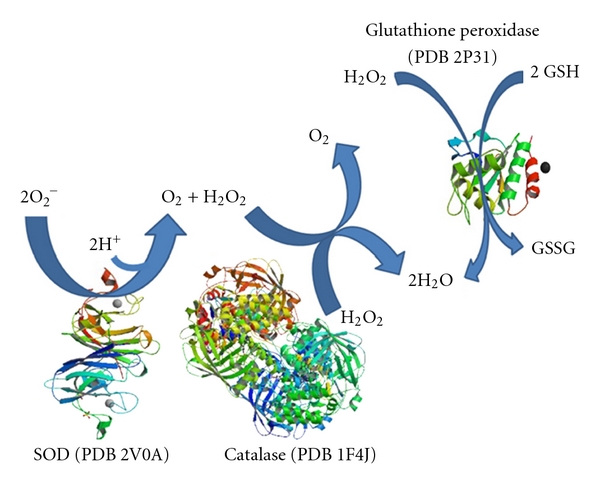
Reactions catalyzed by superoxide dismutase (SOD), catalase, and glutathione peroxidases. Superoxide dismutases (SOD) convert O_2_^−^ to O_2_ and H_2_O_2_. Catalase and glutathione peroxidases convert H_2_O_2_ to water. Protein structures represented in this figure are available in the Protein Data Bank (PDB; http://www.rcsb.org/pdb/home/home.do). PDB ID, are referred in brackets.

Superoxide dismutases (SOD, EC 1.15.1.1) in the mitochondria (Mn-SOD) and cytoplasm (Cu/Zn-SOD) convert O_2_^−^ to oxygen (O_2_) and H_2_O_2_ (Figure 1). In turn, catalase (EC 1.11.1.6) and glutathione peroxidases (EC 1.11.1.9) convert H_2_O_2_ to water (Figure 1). These enzymes are critical for preventing oxidative damages to cells. Thioredoxin and glutathione thiol-based reducing systems are important reductants of many oxidative stressors such as peroxides [14]. Therefore, a biological approach to investigate whether new drugs have molecular mechanism of actions targeted on antioxidant enzymes seems to be an interesting strategy in drug discovery. In addition, other antioxidant molecules, such as *α*-tocopherol (vitamin E) improved the neurological performance and brain mitochondrial functions in mice by presumably decreasing mitochondrial protein and lipid oxidation products [7]. Melatonin has also shown antioxidant properties, since it reduced the levels of malondialdehyde (MDA) plus 4-hydoxyalkenals, which are markers of lipid peroxidation, in the hippocampus, cortex, and cerebellum of rats exposed to 3000 ppm thinner fumes (containing 66% toluene) [15]. 

**Figure 2:**
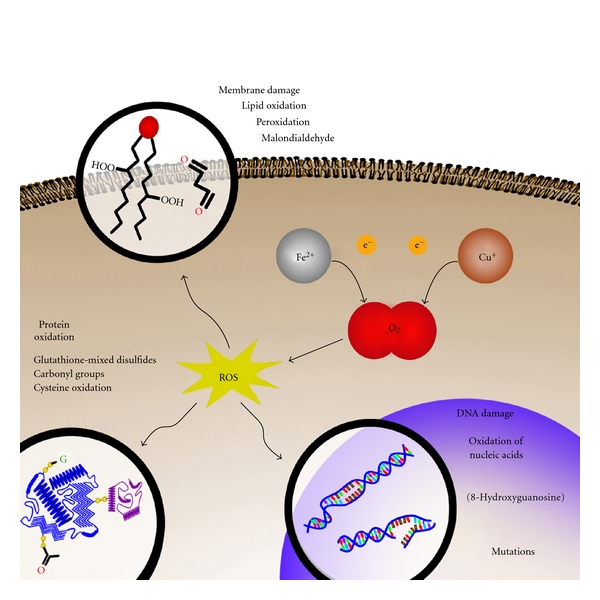
Oxidative damages induced by transition metals. Iron and copper can reduce oxygen leading to ROS generation and subsequent oxidation of proteins, lipids, and nucleic acids.

Mitochondria are not the only source of ROS involved in neurodegeneration. Transition metal ions are capable of stimulating free radical formation, and markers of OS (Figure 2) precede pathologic lesions in AD, including senile plaques and neurofibrillary tangle [16]. The main features of enhanced OS in the AD brain involve increased content of reduced copper and iron capable of stimulating free radical generation, increased carbonyls, protein and DNA oxidation, increased MDA and enhanced lipid peroxidation, decreased levels of cytochrome c oxidase, increased advanced glycation end products, ONOO^−^, and heme oxygenase-1 [17]. The measure of carbonyl content in protein lysates from brain microvessels isolated from rats showed an increase in vascular OS with age [18]. Studies performed on *postmortem* substantia nigra from PD patients demonstrated increased levels of reduced iron and MAO-B activity, OS, inflammatory processes, glutamatergic excitotoxicity, NO synthesis, abnormal protein folding and aggregation, reduced expression of trophic factors, depletion of GSH, and altered calcium homeostasis [5]. It has also been suggested that iron accumulation may contribute to the OS-induced apoptosis reported in both PD and PD dementia [19]. Furthermore, ATP depletion or lipid and protein peroxidation induced by ROS are also implicated in PD and can kill neurons by necrotic processes [13]. Therefore, transition metals have been selected as targets for the research and development of new drugs.

**Figure 3:**
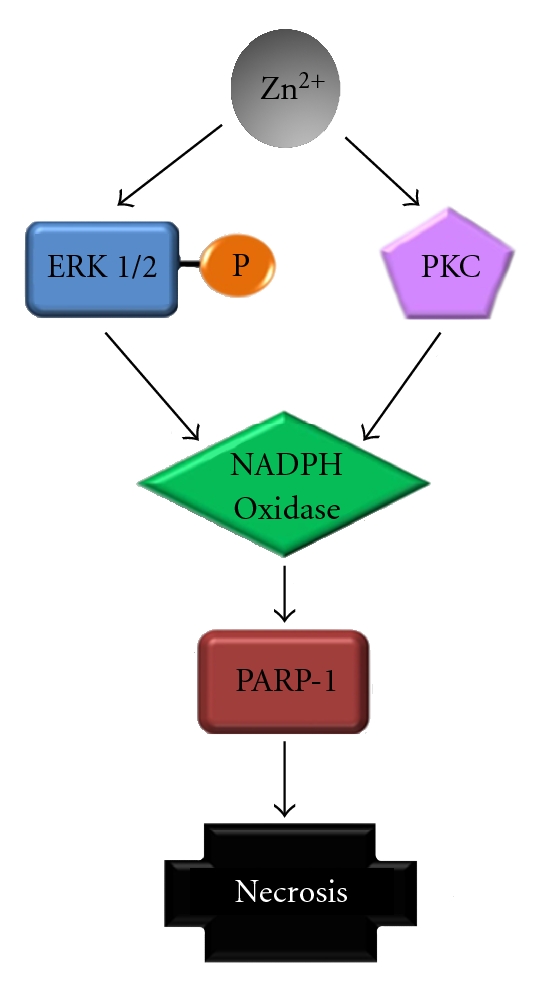
Cell death induced by zinc. Zinc modulates protein kinases, which activates NADPH oxidase and PARP-1, leading to necrosis.

In AD, the amyloid-*β* peptide (A*β*) is released into the glutamatergic synapse cleft where it has the potential to react with copper and zinc to form oxidized, crosslinked soluble aggregates, and precipitated amyloid [20]. Although in the central nervous system (CNS) Zn^2+^ is a component of proteins involved in the defense against OS [21] and is implicated in the regulation of channels and receptors, this cation can also act as a trigger for neuronal loss in several neurological conditions linked to mitochondrial dysfunction and OS [22]. A major physiological role for Zn^2+^ may be the modulation of cell signaling cascades, especially those involving protein phosphorylation [23]. The autophagy is reduced or blocked in neurodegenerative conditions, including AD, PD, HD, and Niemann-Pick type C disease. This is probably modulated by zinc and metallothionein 3, associated with activation of protein kinase C (PKC, EC 2.7.11.13), NADPH oxidases (EC 1.6.3.1), extracellular-signal-regulated kinase (ERK) 1/2 (EC 2.7.11.24), and poly ADP-ribose polymerase (PARP, EC 2.4.2.30), causing oxidative neural necrosis [24] (Figure 3). Heavy metals with no biological function, such as mercury, are also potentially harmful to the human brain. Mercury is a widespread natural and anthropogenic environmental contaminant. Even though the mechanisms underlying mercury toxicity are still unclear, it lowered the catalase activity and increased the glutathione reductase (EC 1.8.1.7) activity in brain homogenates of contaminated fishes (*Dicentrarchus labrax*) [25]. However, there is no established correlation between ND and exposure to heavy metals.

Proteins are one of the prime targets for oxidative damage, and cysteine residues are particularly sensitive to reversible and irreversible oxidation [26]. The level of protein-glutathione-mixed disulfides is increased in the brain of old rats, which represents a reliable index of thiol-directed OS [27]. Although apomorphine is a potent dopaminergic agonist largely used in the treatment of PD, it covalently binds to proteins, probably in cysteine residues, because this amino acid prevented the formation of adducts [28]. The pathologic interaction of cerebral A*β* with transition metals such as zinc, copper, or iron is possibly a neurochemical factor that initiates A*β* deposition [29]. The toxic effects that stem from the misassembly or aggregation of proteins or peptides are collectively termed proteotoxicity [26]. A deregulation of brain iron and cooper homeostasis is a key factor to early neuropathological events in AD, including OS, inflammatory processes, proteotoxicity, tau phosphorilation, and neuronal cell cycle regulatory fail, leading to apoptosis [21]. Glyceraldehyde-3-phosphate dehydrogenase (GAPDH, EC 1.2.1.12) may also be a target, since massive OS caused *in vivo* by methamphetamine induces the formation of GAPDH aggregates in mouse brain [30]. Proteins that appear to have a key role in disease pathogenesis constitute targets for drug discovery.

**Figure 4:**
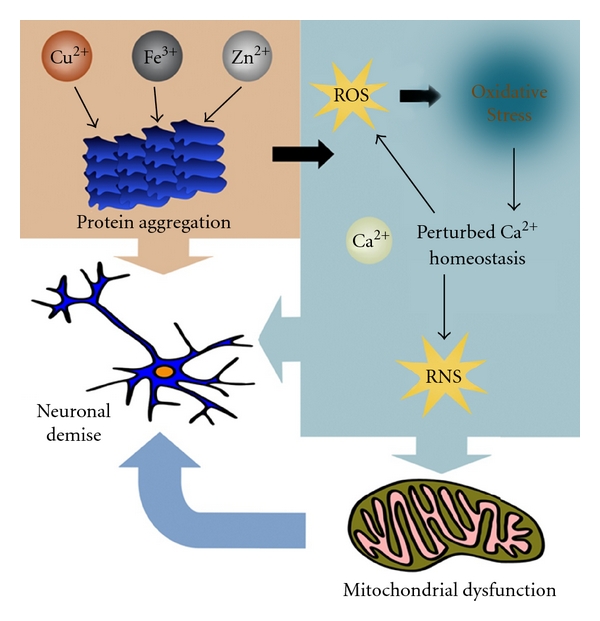
Neuronal demise in Alzheimer's disease. The pathologic interaction of cerebral A*β* with transition metals induces oxidative stress, perturbed cellular Ca^2+^ homeostasis, and energy metabolism, which in turns generates more ROS leading to neuronal demise.

Oxidative stress, perturbed energy metabolism, and alterations of disease-related proteins result in Ca^2+^-dependent synaptic dysfunction, impaired plasticity, and neuronal demise [31]. The causes and consequences of OS in neurons in AD are not fully understood, but considerable evidence points to important roles for accumulation of A*β* upstream of OS, and perturbed cellular Ca^2+^ homeostasis and energy metabolism downstream of it [32] (Figure 4). On the other hand, increased intracellular Ca^2+^ would lead to an increase in mitochondria-derived ROS and RNS generation by disturbing the respiratory chain and activating a series of enzymes, including neuronal NO synthases (EC 1.14.13.39) and xanthine oxidases (EC 1.17.3.2), which catalyze ROS or RNS formation by triggering the arachidonic acid cascade [33]. NO activates the epsilon isoform of PKC, which in turn activates the Src family of protein tyrosine kinases and transcription factors with resultant upregulation of expression of many genes and proteins [34]. Curiously, age-related macular degeneration, which is a late-onset, neurodegenerative retinal disease, shares several clinical and pathological features with AD, including stress stimuli such as OS and inflammation [35]. Oxidative stress, protein aggregation and redox active metal ions can all be considered potential targets for the treatment of neurodegeneration. 

Although normal excitatory neurotransmission is essential for synaptic development and plasticity as well as learning and memory, the excessive stimulation of glutamatergic receptors induces neurotoxicity and plays a role in ND. It is known that the excitatory amino acid *β-N-*oxalyl-*α*,*β*-diaminopropionic acid binds to *α*-amino-3-hydroxy-5-methyl-4-isoxazole propionic acid (AMPA) receptors triggering motor neuron degeneration by inducing OS and cell death [36]. The excessive nitrosative and OS that results from the hyperactivation of the AMPA-type glutamate receptors is thought to trigger cellular signaling pathways leading to neurodegenerative conditions [37]. Modifications in excitatory glutamatergic neurotransmission provide a rational basis for the discovery of new medicines. 

**Figure 5:**
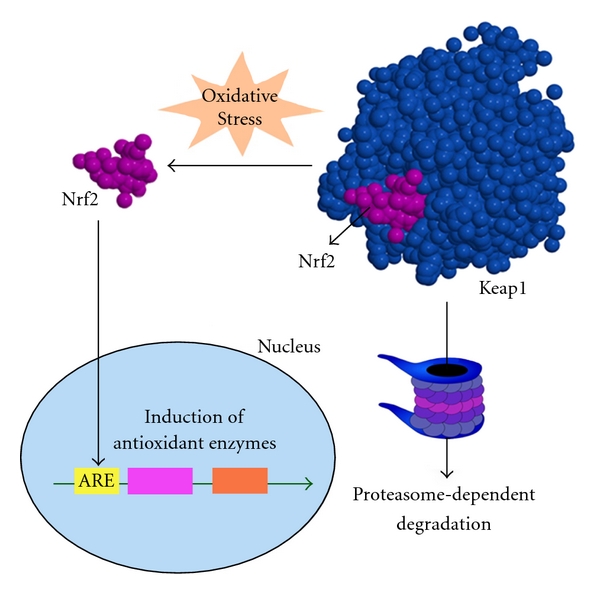
Nrf2 regulates the expression of antioxidant enzymes. Under physiological conditions, Nrf2 binds to Keap1 and undergoes constitutive proteasome-dependent degradation. However, under oxidative stress, Keap1 changes its conformation and releases Nrf2, which moves to the nucleus and binds to ARE regulating the expression of several antioxidant enzymes.

Among the several environmental factors proposed for AD, dietary risk and protective factors have been most compelling. Diets rich in saturated fatty acids, alcohol, and deficient in antioxidants and vitamins appear to promote the onset of the disease, while diets rich in unsaturated fatty acids, vitamins, antioxidants, and wine likely suppress its onset [38]. Actually, there is a great interest to study the neuroprotective effects of natural products obtained from plants. The major lignans contained in sesame seeds are sesamin and episesamin. (1*R*,2*S*,5*R*,6*S*)-6-(3,4-dihydroxyphenyl)-2-(3,4-methylenedioxyphenyl)-3,7-dioxabicyclo-[3,3, 0]octane (SC-1), (1*R*,2*S*,5*R*,6*S*)-2,6-bis(3,4-dihydroxyphenyl)-3,7-dioxabicyclo-[3,3, 0]octane (SC-2), and (1*R*,2*S*,5*R*,6*R*)- and (1*R*,2*R*,5*R*,6*S*)-6-(3,4-dihydroxyphenyl)-2-(3,4-methylenedioxyphenyl)-3,7-dioxabicyclo-[3,3, 0]octane (EC-1) are derivatives of sesamin and episesamin that activated the nuclear factor-erythroid-2-related factor 2 (Nrf2)/antioxidant response element (ARE) [39]. Nrf2 protein is a Cap'n'Collar basic-region leucine zipper transcription factor responsible for activating transcription of more than 100 known, mainly cytoprotective genes—the so-called “Nrf2 battery” [40]. The activation of Nrf2/ARE regulates the expression of antioxidant enzymes (Figure 5). Under unstressed conditions, Nrf2 binds to Kelch-like ECH-associated protein 1 (Keap1) and undergoes constitutive proteasome-dependent degradation. In the presence of OS, Keap1 changes its conformation and releases Nrf2, which moves to the nucleus and binds to ARE. The induction of antioxidant enzymes by activation of Nrf2/ARE signaling has been considered as a promising strategy to fight against OS-related diseases. The expression and/or activation of Nrf2 in neurons can be strongly protective [14] and provides an interesting biological hypothesis to be tested and a starting point for the identification of molecules to do this.

## 3. Looking for New Neuroprotectants

Since there is no effective drug in the control of ND, in the next paragraphs some recent data on drug screening for neuroprotectants were reviewed. Tanshinone IIA, one of the key components of *Salvia miltiorrhiza *Bunge roots, protected primary rat cortical neurons against H_2_O_2_-induced cytotoxicity *in vitro *[41]. The extract of *Lonicera japonica *Thumb., which is a shrub traditionally used in Korea and China, also significantly inhibited H_2_O_2_-induced SH-SY5Y human neuroblastoma cell death [42]. Hydrogen peroxide induces the phosphorylation of the protein kinase Akt and inactivates ERK phosphorylation. Prosaposin, a multifunctional protein with versatile neurotrophic activities, which is found in the cerebrospinal fluid, rescued PC12 cells from H_2_O_2_-induced cytotoxicity through the regulation of Akt and ERK phosphorylation pathways [43]. The ethanol extract obtained from leaves of the *Eriobotrya japonica*, which is a traditional medicinal plant in East Asia, protected rat pheochromocytoma PC12 cells against A*β*_1–42_-induced cytotoxicity and formation of intracellular ROS [44]. In these different *in vitro* models, cells were severely injured with agents that promptly induce fatal cellular damages. However, it is not known if the protective effects of these agents observed *in vitro* will provide a sufficient therapeutic index *in vivo*.

Synthetic compounds have also been tested. 2-Ethoxy-4,5-diphenyl-1,3-oxazyne-6-one presented neuroprotective effects against H_2_O_2_-induced PC12 cell death [45]. Another reported neuroprotective agent is 5-chloro-7-iodoquinolin-8-ol (clioquinol), which is an effective iron chelator that blocked the formation of H_2_O_2_ induced by A*β* [21]. However, since halogenated hydroxyquinolines possess neurotoxic side effects, highly selective iron(III) hydroxypyridinone-based ligands have been synthesized in a range of lipophilicity to permit good blood-brain barrier (BBB) permeability [46]. The characteristic of these new molecular entities needs to be analyzed.

Actually, obesity is an important health care issue. There is a growing appreciation that the complications of obesity extend to the CNS. Deficits in a learning task that is dependent at least in part on hippocampal function were observed in rats with obesity phenotype elicited by downregulation of hypothalamic insulin receptor [47]. Furthermore, in a case-control analysis, in which participants were drawn from the Swedish Twin Registry, overweight and obesity at midlife were related to dementia with odds ratios (95% CI) of 1.71 (1.30–2.25) and 3.88 (2.12–7.11), respectively [48]. 

In 16-month-old male C57BL/6 strain mice, which received 2% cholesterol/daily by intraperitoneal injection for 16 weeks, a significant increase in ROS was measured in brain homogenates compared to control animals that did not receive it, but interestingly, it was attenuated by daily oral administration of the flavonoid quercetin at doses of 60 mg·Kg^−1^·day^−1^ [49]. Dietary polyphenols, such as flavonoids, are derived from plants and are consumed in the forms of fruits, vegetables, and wine. Baicalin is a flavonoid that is a component of *Scutellaria baicalensis *Georgi roots, which have been widely used in China to treat stroke for thousands of years. This compound protected human SH-SY5Y neuroblastoma cells against A*β*-induced cytotoxicity and H_2_O_2_ production [50]. Resveratrol is the most outstanding representative of wine polyphenols. Resveratrol has neuroprotective features in both *in vitro* and *in vivo* models of AD, ischemic stroke, PD, HD, and epilepsy [9]. It has been argued that many of the neuroprotective actions in animal models that are ascribed to natural botanical antioxidants, such as flavonoids, polyphenols, and tocopherols actually derive from the pharmacological antagonism by these compounds of enzyme systems that produce reactive oxidant species [51]. Quercetin and resveratrol activate the Nrf2/Keap1 signaling pathway [40]. 

The pesticide rotenone is a naturally occurring plant compound commonly used in vegetable gardens as insecticide. Neurons exposed to rotenone (0.3 *μ*M) generated O_2_^−^, an effect that was inhibited by *N-*acetylcysteine [52]. The rotenone-treated human dopaminergic cells SH-SY5Y are a suitable model for investigating new possible therapeutic targets for PD. *Rhus verniciflua *Stokes of the Anacardiaceae family is commonly known as lacquer tree. The clinical application of this plant has been limited because an allergenic mixture of several derivatives of catechol, called urushiol, causes severe contact dermatitis. However, it can be removed during the extraction process. The pretreatment of SH-SY5Y cells with the detoxified extract obtained from the bark of this plant significantly diminished rotenone-induced ROS formation [53]. Molecules bearing the catechol group may be neurotoxic by inducing OS depending on the concentration. The simplest catechol molecule, 1,2-dihydroxybenzene, which is a benzene metabolite, killed neuroblastoma N2a cells after 72 hours, when treated with concentrations of at least 20 *μ*M, and presented an EC_50_ of 38 *μ*M, an effect that was significantly prevented by L-cysteine [54]. The presence of a catechol group in a molecule can interfere in both drug efficacy and safety.

The histone deacetylase (EC 3.5.1.98) of the family 6, which catalyzes the deacetylation of lysine residues on histone and several other proteins such as *α*-tubulin, is another potential therapeutic target. It seems that this enzyme is involved in some ND, since its selective inhibition by mercaptoacetamides protected neurons against the OS-induced neurodegeneration [55]. This suggests that the molecular mechanism of action based in the interaction between drugs and this enzyme should provide a therapeutic effect.

## 4. Is There Any Relationship between Genes and Oxidative Stress?

The term gerontogenes has been suggested to refer to any genetic elements that are involved in aging [56]. Since it is necessary to look for genes as the ultimate controllers of all biological processes, it is important to identify gerontogenes involved in ND. Furthermore, the maintenance of optimal long-term health conditions is accomplished by a complex network of longevity assurance processes which are controlled by vitagenes, such as those that encode heat-shock proteins, thioredoxin, and the sirtuin protein systems [26]. This group of genes preserves cellular homeostasis during stressful conditions. Omega-3 polyunsaturated fatty acids have the ability to regulate several genes associated with OS, apoptosis, as well as cell signaling and division, and may constitute a therapeutic strategy for several CNS disorders, although further clinical trials are needed to investigate it [57].

Although most cases of AD are sporadic, 5–10% of AD patients suffer from familial AD with an autosomal dominant inheritance pattern. However, despite the overexpression of A*β*_1–42_ from birth, it is not the solely responsible factor for amyloid deposition, because it is not observed in childhood. Zinc, copper, and iron have been implicated as initiating factors due to high concentration gradients of these metals in the cortex, hippocampus, and the cortical vasculature—brain regions that are severely affected by the pathological lesions of AD [29]. 

The human MAO-A and MAO-B promoters cloned into the luciferase reporter construct pGL3 were activated when cotransfected with the nuclear orphan estrogen-related receptor *α* in HeLa cells, but it was almost completely abolished by the cotransfection with the parkin gene [58]. This means that parkin may control OS by limiting the expression of MAO, responsible for the oxidative deamination of dopamine. Furthermore, upregulation of parkin can compensate for neuronal loss caused by *PINK1* mutations at least in flies, but this mutation is also seen in cells from PD patients [59].

HD is a fatal autosomal dominant neurodegenerative disorder of midlife onset involving the protein huntingtin (Htt) that is expressed widely and heterogeneously in neurons throughout the brain. This genetic disease is associated with the degeneration of GABAergic striatal projection neurons in the basal ganglia leading to movement disorders with behavioral symptoms for which there is presently no therapy. However, a direct pathway linking the genetic mutation to neuronal degeneration has not been established. In PC12 cells modified to express mutant Htt, there was a caspase 3 (EC 3.4.22.56) activation, which was reduced by an indolocarbazole analog (CEP-1347) [60]. The polyphenol fisetin also increased the survival of mutant PC12 cells [61]. Furthermore, other genes seem to be regulated in HD cases like MAO-A, which was found significantly increased throughout the caudate and putamen, whereas MAO-B was even higher compared to controls [62]. Therefore, MAO seems to be involved in the induction of OS and lipid peroxidation. 4-Hydroxy-2-nonenal (HNE), a highly reactive lipid peroxidation product, is increased in caudate and putamen of human HD brains [63]. HNE can irreversibly modify proteins by binding covalently to cysteine, lysine, and histidine residues [64]. Furthermore, under OS and in the presence of NO, GAPDH undergoes posttranslational modifications, especially *S*-nitrosylation. The modified GAPDH is translocated to the nucleus upon exposure to stressors and participates in cell death. GAPDH has been found in nuclei of fibroblasts and in postmortem brains from patients with HD [30].

MJD/spinocerebellar ataxia type 3 is an autosomal dominant spinocerebellar degeneration caused by polyglutamine expansion in the ataxin-3 protein. It is characterized by cerebellar ataxia and pyramidal signs associated in varying degrees with a dystonic-rigid extrapyramidal syndrome or peripheral amyotrophy as major neurologic signs. Human neuroblastoma SK-N-SH cells transfected with the mutant ataxin-3 presented lower total glutathione and GSH contents than the wild type [6]. Furthermore, there was a decrease in activities of glutathione reductase, catalase, and superoxide dismutase, which suggests that these modified cells are prone to damages under OS.

Although neurodegenerative disorders have a more common late-onset, in some rare genetic diseases, such as juvenile neuronal ceroid lipofuscinosis (Batten disease), the neurodegeneration has an early onset, usually at 5–7 years. Even in this juvenile neurodegenerative disease, OS is involved in the pathological mechanism. Batten disease is caused by mutations in the *CLN3* gene that encodes a highly hydrophobic, multispanning transmembrane protein of unknown function. In a *CLN3 Drosophila* mutant model, mutant flies were more susceptible to OS induced by H_2_O_2_, diethylmaleate, which depletes GSH, and paraquat, which generates O_2_^−^ [65]. Furthermore, since the overexpression of *CLN3* conferred resistance to OS in flies, this gene might be used in vectors [66], as a strategy that could increase its expression in ND. 

The adaptation and survival of cells and organisms requires the ability to sense proteotoxic insults and to coordinate protective cellular stress response pathways and chaperone networks related to protein quality control and stability [26]. Inherited Friedreich ataxia (FRDA) is a neurodegenerative disease characterized by a mutation of the frataxin gene with disturbed iron function. This protein serves as a chaperone in iron-sulphur cluster assembly, but the mutation leads to mitochondrial free iron overload and ROS formation [67]. *α*-Tocotrienol quinone seems to be protective in this disease, because it rescued primary fibroblasts from an FRDA patient from cell death induced by L-buthionine-(*S,R*)-sulfoximine, which depletes GSH [68]. Ongoing FDA-approved, open-label human studies in patients with inherited mitochondrial disease receiving *α*-tocotrienol quinone at doses between 100–400 mg three times daily, no significant elevation of clotting times or significant drug-related adverse events have been reported [68].

Therefore, in the main ND, several genes are related to mechanisms that generate ROS. On the other hand, some genes seem to be important in the protections of neurons against ROS, since neurodegeneration occurs when there are mutations. It is important to know the involvement of genes in neurodegeneration and in neuroprotection because they can be targets for therapeutic approaches.

## 5. The Role of Glial Cells

Neurons depend on glial cells to survive during all life, and it is important to understand the functioning of these cells in aging and ND. The glial cell line-derived neurotrophic factor (GDNF) seems to play a physiological role in the maintenance and function of the aging nigrostriatal system [69]. Riluzole induced GDNF mRNA expression and release via fibroblast growth factor receptor signaling in rat C6 glioma cells, which are used as a model of astrocytes [70]. In addition, Nrf2 overexpressing transfected glial cells strongly protected surrounding untransfected neurons [14]. The treatment of C6 cells with 6-hydroxydopamine increased the nuclear translocation and transcriptional activity of Nrf2, which seemed to be partly mediated by activation of upstream kinases, such as Akt/protein kinase B (EC 2.7.10.2) [71]. As it was stated before, Nrf2 regulates the expression of several antioxidant enzymes.

Astrocytes express several enzymes of drug metabolism, which protect neurons against reactive xenobiotics. Rat brain microsomes were able to catalyze the glucuronidation of planar phenols [72]. Astrocytes keep glutamate and ammonia at low levels in the CNS because these cells express glutamate-ammonia ligase, more commonly known as glutamine synthetase (GS; EC 6.3.1.2). However, OS and iron levels in ND may interfere in the activity of this enzyme. The GS activity in mouse astrocyte primary cultures is inhibited by H_2_O_2_ at high concentrations, an effect that was reverted by iron chelators such as 1,10-phenanthroline and 2,2′-dipyridyl [73]. In addition, MAO-B is expressed in glial cells and it can contribute to ROS generation. Cellular iron and MAO levels are elevated in the aging brain and in brains burdened with ND because of gliosis [74]. 

Activated glial cells are histopathological hallmarks of ND. In response to neuronal injury, microglia and astrocytes become activated and secrete inflammation mediators such as cytokines, chemokines, and the potentially damaging NO and ROS [34]. PD is associated with neuroinflammation, in which the hallmarks are the presence of activated microglia and reactive astrocytes in the parenchyma of the CNS, and increased production of ROS and RNS that in some case can result in disruption of the BBB [75]. Although flavonoids have been adopted in popular medicine as antioxidants, rutin is able to activate microglial cells *in vitro* at concentrations above 50 *μ*M [76]. Alkaloids can also activate glial cells *in vitro* and stimulate NO production [77]. The activation of glial cells can be deleterious to neurons. The bacterial endotoxin lipopolysaccharide causes dopaminergic neurodegeneration via activation of surrounding glial (mainly microglial) cells [69]. HNE, which results from lipid peroxidation, upregulates cytosolic phospholipase A_2_ (PLA_2_) and its phosphorylated activated form in cultured Ra2 microglial cells [78]. PLA_2_ is a well-known proinflammatory enzyme. The generation of ROS is not only deleterious to neurons. Oligodendroglial cells are also highly susceptible to oxidative damage. These cells have a high metabolic rate with toxic by-products, an increased requirement for ATP, high intracellular iron level, and low concentrations of GSH [79]. Although glial cells are physiologically very important for neurons, these data show that in pathological conditions their activation can lead no neuron demise. It is important to understand the role of glial cells to neurodegeneration because they can be targets for the development of new drugs and therapeutical strategies.

## 6. What Are the Main Advances in Therapeutics?

Current therapeutic strategies in ND have focused mainly on alleviating symptoms and improving quality of life. A multifunctional treatment, with symptomatic and neuroprotective properties, would be ideal to the patient, as the probability of side effects due to drug interactions would be reduced, besides being more convenient [19]. Furthermore, mounting evidence in the peer-reviewed literature shows that the etiopathology of these diseases is extremely complex and heterogeneous, resulting in significant comorbidity and, therefore, unlikely to be mitigated by any drug acting on a single pathway or target [74]. The primary objective of neuroprotective therapies is to prevent neuronal degeneration by interfering in the underlying neurodegenerative mechanisms that result in cell dysfunction and death [59]. It is important to stress that a drug can have neuroprotective properties, but be ineffective on neural restoration or neuro rescue, as these effects are accomplished by different mechanisms of action. Ideally, neuroprotective therapies should provide some protection against neurotoxins, ROS, and free radicals, besides promoting neuronal survival through the upregulation of neurotrophic factors [5]. However, the involvement of multiple pathways plus the absence of a reliable biomarker to assess disease progression makes the evaluation of potential neuroprotective treatments difficult. Current animal models do not replicate the disease process, such as in PD, and there is no validated quantitative biomarker to assess disease progression in patients. In the absence of a reliable biomarker of disease progression, some clinical trial strategies were developed in order to evaluate neuroprotective effects of some potential drugs. As the majority of these drugs are used to treat symptomatically patients, and clinical trials do not assess mechanisms of action, neuroprotective results may be confounded by potential symptomatic effects of these medications.

## 7. MAO-B Inhibitors

Neurons of the substantia nigra pars compacta are more susceptible to OS due to dopamine, melanin, and iron concentration. In addition, the dopamine metabolism by MAO-B leads to formation of H_2_O_2_, which interacts with iron and produces HO^∙^. Decreased levels of GSH were found in autopsy brains of PD patients [5], enhancing the role of OS in neuronal death. MAO-B inhibition is associated with reduction on dopamine turnover and OS. In this context, a randomized placebo-controlled trial called DATATOP (Deprenyl [selegiline] And Tocopherol Antioxidant Therapy Of PD) was developed to evaluate selegiline and Vitamin E as potential disease-modification therapies. PD patients in early stages were randomly assigned to three groups: placebo, Vitamin E (2000 IU) or selegiline (5 mg BID), a MAO-B inhibitor. The primary endpoint was requirement of levodopa therapy. The results showed that patients treated with selegiline had a significant lower risk of reaching the endpoint (26% in the selegiline group and 47% in the placebo group). However, Vitamin E produced no benefits and there was no interaction between the two drugs [80]. Initially, these findings were considered as a potential disease modification property of selegiline. However, at that time it was not considered that selegiline could have a small symptomatic effect responsible for these results. In order to solve these conflicts, a study called SINDEPAR (Sinemet-Deprenyl-Parlodel) was conducted in patients with early PD, designed with a washout period to clear symptomatic effects of the drug and then prove neuroprotection [81]. In this trial, patients were randomly assigned to treatment with selegiline or placebo, or to symptomatic treatment with bromocriptine or levodopa. Patients were followed for 14 months, and selegiline was stopped 2 months prior to endpoint. In addition, bromocriptine and levodopa were stopped a week prior to endpoint. The primary endpoint was the change in motor unified Parkinson's disease rating scale (UPDRS) scores between untreated baseline and final visit, 2 months after a period of selegiline withdrawal. The results showed that patients treated with selegiline had lesser deterioration on UPDRS scores than the placebo group, regardless of their symptomatic treatment (levodopa or bromocriptine). Despite these results, the doubt whether 2 months off selegiline is enough to get rid of all the symptomatic benefits still remains. Considering the drug mechanisms of action, it takes the brain about a month to turn over half of its MAO-B content. Therefore, it is possible that a protracted symptomatic effect could be still present in this clinical trial. 

*N-*propargyl-1-(*R*)-aminoindan (rasagiline) is another example of a MAO-B-targeting drug for use in ND. The primary effect of rasagiline in PD is exerted by MAO-B inhibition, thus leading to clearly symptomatic benefits through the reduced metabolism of endogenous and exogenous dopamine. Rasagiline has shown neuroprotective effects in ethanol-induced cell death mediated by a novel GAPDH-MAO-B pathway [30]. The levels of cholesterol-oxidized products and the depletion of GSH significantly decreased in the striatum of rats treated with rasagiline (0.05 mg/kg daily for 14 days) [82]. Although the evaluation of carcinogenicity studies of rasagiline was positive in mice, which developed lung adenoma and carcinoma, it is not relevant to humans according to the European Public Assessment Reports (EPAR) due to a high safety margin, since tumours were observed at systemic exposures 144–213 times the expected plasma levels in humans [83]. 

A delayed-start design was developed to assess the neuroprotective properties of interventions by separating symptomatic from disease-modifying effects. Studies with delayed-start design are developed in two phases: initially, the groups are randomly assigned to receive either active drug or placebo for a period, followed by a phase in which all groups receive the drug. The differences in the evolution of early versus late onset of therapy are considered as disease-modifying effects exerted by the drug. Two studies, designed to overcome the symptomatic effects of rasagiline were included in this paper. 

The TEMPO study (Rasagiline Mesylate [TVP-1012] in Early Monotherapy for Parkinson's Disease Outpatients) was a randomized, double-blind, placebo-controlled trial designed to evaluate the effects of early versus late rasagiline use on progression of disability in PD patients [84]. This study was carried out during one year with early-stage PD patients who required no dopaminergic therapy, enrolled at 32 centers in the United States and Canada. In the first phase, lasting six months, 404 subjects were randomly assigned to receive rasagiline (1 or 2 mg/daily) or placebo. In the second phase of treatment, patients who were on placebo entered the active phase and received rasagiline (2 mg/daily) for 6 months. The study compared the effects of early versus late rasagiline on PD progression. The primary endpoint was change in total UPDRS score from baseline to 52 weeks later. Secondary endpoints were the proportion of subjects whose UPDRS score decreased by fewer than 4 units during the study (classified as responders), change in the UPDRS motor subscore, activities of daily living (ADL) subscore, and the Beck Depression Inventory score. In a 26-week analysis, both groups receiving rasagiline showed an improvement in the mean UPDRS total score (1 mg: 4.2 points more than placebo and 2 mg: 3.5 points more than the placebo; *P* < 0.00001). In a posterior analysis performed at week 52, mean changes in scores of total UPDRS score from baseline were 3.01 (SD 8.26), 1.97 (SD 7.49), and 4.17 (SD 8.83), in groups treated with 1 mg rasagiline, 2 mg and late onset of 2 mg, respectively. The treatment effect on the UPDRS total score was evaluated by the difference in adjusted means of model analysis of covariance. The comparison between the 1 mg group and the late onset 2 mg group was −1.82 units (95% CI −3.64 to 0.01, *P* = 0.05). The comparison between the 2 mg group with the late onset 2 mg group resulted in −2.29 units (95% CI −4.11 to −0.48, *P* = 0.01). In the efficacy cohort, the percentages of individuals who were considered as responders were 63.8% in the 2 mg rasagiline for 1 year, 52.5% in the 1 mg rasagiline for 1 year, and 52.3% in the 2 mg rasagiline delayed-start. Although statistically significant, a difference of just 1% in the UPDRS scale must be cautiously interpreted as a clinical improvement.

The ADAGIO study (Attenuation of Disease Progression with Azilect Given Once-daily) was a randomized, double-blind, placebo-controlled, multicentre study carried out in 1176 untreated PD subjects in early stages of the disease, during 72 weeks to evaluate whether the rasagiline therapy slows the disease progression [85]. This study was conducted in two phases, each one lasting 36 weeks. At first, subjects were randomly assigned into four groups to receive 1 or 2 mg rasagiline/day, or corresponding placebo. In the second phase, the subjects on the placebo group initiated the active treatment phase, starting rasagiline (1 or 2 mg/day; late onset), while the others continued to receive the doses previously established. The primary analysis consisted of three hierarchical end points that assessed changes from baseline in the total UPDRS score sections I through III. The first end point was measured from weeks 12 through 36 and showed a slower progression rate in the rasagiline group treated with 1 mg/day (0.09 ± 0.02 points per week), when compared to placebo (0.14 ± 0.01 points per week; *P* = 0.01). The second end point, assessed between baseline and week 72, showed that the early-start group had less worsening in the mean total UPDRS score when compared to the late-onset group (2.82 ± 0.53 versus 4.50 ± 0.56 points; *P* = 0.02). The third end point (week 48 through 72) showed the noninferiority of the estimated changes in the total UPDRS score in the early-onset group when compared to the late-onset group (0.085 ± 0.02 versus 0.085 ± 0.02 points per week; *P* < 0.001). Among subjects receiving rasagiline (1 mg/day) from the beginning, all three end points previously established were achieved. Among subjects receiving 2 mg rasagiline from the beginning, the rate of disease progression was also lower; however, there was not a statistical significant difference in total UPDRS score when compared to the late-onset group (3.47 ± 0.50 points versus 3.11 ± 0.50, *P* = 0.60). 

Although it seems promising at a quick glance, the clinical trials showing neuroprotective effect of rasagiline should be considered with caution. The delayed-start trial design has several limitations that might interfere with study results, such as the nonlinear progression of PD, the possibility that all symptomatic drugs could produce positive results in delayed start trials, the fragility of the UPDRS in assessing the outcomes, and the poor generalizability of results due to the selection of slower disease progression patients. The different results observed for the two doses of rasagiline are difficult to explain, but it is possible that a higher dose of rasagiline may have preponderant symptomatic effect and masked a possible disease-modifying effect in PD subjects with milder disease. Another explanation is that neuroprotection can follow a U-shaped curve, which could theoretically account for the failure of the 2 mg dose [86]. Furthermore, the clinical meaning of a difference of just 1.7 units in the UPDRS score is also questionable. It is clear that further studies addressing the effects of rasagiline on disease progression in PD patients are required to support the positive findings for the 1 mg dose of rasagiline. The inclusion of subjects in a more advanced stage of the disease could help to differentiate the symptomatic from the neuroprotective effects in higher doses of rasagiline. In addition, a longer following up period would provide data on the persistent benefits of the early-start rasagiline on disease progression. At this moment, it is not possible to establish the neuroprotective effect of rasagiline in PD patients using the existing evidence. However, even if someone consider the crude results of these clinical trials, it is hard to convince someone that just 1% of clinical improvement is sufficient to affirm the so-called neuroprotective property of rasagiline. A recent trend in drug design and discovery is the rational design or serendipitous discovery of novel drug entities with the ability to address multiple drug targets that form part of the complex pathophysiology of a particular disease state [74]. Ladostigil is a multifunctional compound designed to incorporate the cognitive enhancing properties of rivastigmine with the neuroprotection properties of rasagiline [87]. However, it was not tested in clinical trials, yet. 

Lazabemide is a short-acting and reversible inhibitor of MAO-B that is not metabolized into amphetamines. In a randomized double-blinded and placebo-controlled trial, 321 untreated patients in early stages were assigned into 5 arms (placebo, 25 mg/day, 50 mg/day, 100 mg/day and 200 mg/day) and followed over one year [88]. In this study, the risk of requiring levodopa therapy was reduced by 51% in the lazabemide group when compared to placebo (*P* < 0.001), and the effect was consistent among all dosages. However, similarly to selegiline, lazabemide also have symptomatic properties, which may have masked the results [89]. 

In preclinical models of HD, CEP-1347 showed promising results [60]. Therefore, a phase III double-blinded trial was conducted in 806 early PD patients randomized into four arms (10 mg BID, 25 mg BID, 50 mg BID, or placebo), and the primary endpoint considered was time to levodopa therapy. However, the study was interrupted earlier as there were no significant differences between placebo and CEP-1347 groups in any endpoints considered [90].

## 8. Antiglutamatergic Agents

Preclinical studies have demonstrated the potential capacity of riluzole to protect dopamine neurons [10, 91]. In order to determine if riluzole has neuroprotective properties, a multicenter placebo-controlled study was conducted in untreated patients in early stages of PD. Patients were randomized to receive 50 mg riluzole capsules orally, taken twice daily, or a matching placebo. After the washout period, all patients were offered a one-year open label extension study. Principal endpoints were time required to levodopa therapy and changes in UPDRS scale. Riluzole did not show significant neuroprotective effects in any of the endpoints assessed [92].

## 9. Antioxidants and Vitamins

Substances that delay, prevent, or remove oxidative damages to a target molecule are considered as antioxidants, but efforts to develop an effective antioxidant treatment for ND have remained elusive. Although *Ginkgo biloba* extract protected cells against GSH depletion [12] and vitamin E is an antioxidant, the British Association for Psychopharmacology considered that until further evidence is available, they are not recommended either for the treatment or prevention of AD [93]. Several new compounds cited in this text that were tested in preclinical assays are not yet approved by the U.S. Food and Drug Administration (FDA; http://www.accessdata.fda.gov/scripts/cder/drugsatfda/). This is case for extracts of *L. japonica, E. japonica*, and *R. verniciflua,* the protein prosaposin, the synthetic compound 2-ethoxy-4,5-diphenyl-1,3-oxazyne-6-one, and the flavonoids baicalin, fisetin, and quercetin. Melatonin, which has shown antioxidant properties *in vivo* [15], sesamin, episesamin, and their derivatives that induced antioxidant enzymes by activating Nrf2/ARE signaling are not approved by the FDA, too. However, in a randomized, placebo-controlled, crossover study, involving 24 postmenopausal healthy women, sesame ingestion decreased total cholesterol and thiobarbituric acid reactive substances in LDL, meanwhile it increased the ratio of *α*- and *γ*-tocopherol to total cholesterol [94]. Although the flavonoid resveratrol is not approved by FDA, it is now in early stages of clinical trials, but not for ND, its efficiency has been tested in the cancer treatment [51]. Tanshinone IIA is also not approved by FDA, but emerging experimental studies and clinical trials have demonstrated atherogenesis prevention [95]. 

Actually, the possible clinical uses of vitamins in ND have been studied. The supplementation of vitamin E or C for 3-4 years in men from Hawaii aged between 71 to 93 years with dementia and cognitive dysfunction found that both vitamins improved cognitive performance along with protection only against non-AD dementia [96]. In a metaanalysis of three vitamin E clinical trials, four trials of coenzyme Q10, and 1 study of glutathione, only the coenzyme Q10 trials demonstrated some minor treatment benefits in PD that probably map to partial correction of mitochondrial electron transport chain deficiency [96]. One prospective study failed to find an association between dietary folate, vitamin B12, or vitamin B6 with incident AD [97]. Based on several clinical trials showing that supplementation with folic acid with or without vitamin B12 does not benefit cognition in people with dementia, the British Association for Psychopharmacology does not recommend their use either for dementia treatment or prevention [93]. The possible clinical use of vitamin D has also been investigated. Vitamin D promotes health by regulating autophagy, which is a catabolic process necessary for cells to degrade cytosolic macromolecules and organelles in the lysosome [98]. Few double-blind placebo-controlled prevention trials support the hypothesis that calcipherol hormones are significantly involved in several chronic ND and they could be used in preventive medicine [99]. However, more studies are needed before final conclusions can be made.

## 10. Metal Chelators

Oxidative stress has been a therapy target in clinical trials, but results have largely been negative or mild at best. However, it is important to look for new metal chelators, since in a clinical trial the licensed drug desferrioxamine was found to reduce the rate of progression of AD by a factor of 2 within a two-year period [100]. However, despite evidence showing the involvement of metals in ND, a case-control study in Japan found that a higher intake of iron, magnesium, and zinc was independently associated with a reduced risk of PD [101]. However, a weakness in this study is that control subjects were significantly younger than cases with PD. Among other iron chelators, clioquinol is approved by FDA for topical use as ointment in association with nystatin, but this is no more found on marketing because its production was discontinued. Anyway, it was not approved to be used in ND. Other metal chelators such as 1,10-phenanthroline and 2,2′-dipyridyl that have been used in preclinical tests are not approved by FDA.

## 11. Concluding Remarks

Investment in drug research and development has increased in recent decades, but the annual number of truly innovative new medicines has not increased accordingly, which creates a problem to the pharmaceutical industry to replace the loss of revenues due to patent expirations [102]. Despite evidences that OS plays a role in ND, a good target for pharmacological management rests to be determined. Although ROS should be a therapeutic target in ND, this is necessary to recognize that an antioxidant in chemical systems may be not an efficient agent in biological ones. The effectiveness of antioxidants is probably limited by their bioavailability and the fact that they would have to be present at high concentrations to be able to compete with endogenous targets. Several potential drugs have been screened using *in vitro* and *in vivo* models of ND, but these results were not translated in benefit of patients, and disappointing results were obtained in the majority of clinical trials. However, several potential drugs and their targeted pathways were not yet tested in clinical trials. Among the drugs tested in clinical trials, selegiline and rasagiline are the most promising ones at least in PD, but their neuroprotective effects remain to be better investigated. Even if OS plays a contributory role to the neurodegeneration process, it is likely not the only pathological insult that needs to be targeted.
